# Connecting the Dots: Mass Media, Maternal Exposure, and Child Health Outcomes in Benin

**DOI:** 10.1002/puh2.70026

**Published:** 2025-02-25

**Authors:** Joseph Kawuki, Meroona Gopang, Sylvester R. Okeke, Edward Kwabena Ameyaw, Sanni Yaya

**Affiliations:** ^1^ Program in Public Health Department of Family, Population, & Preventive Medicine Stony Brook University Stony Brook New York USA; ^2^ Centre for Social Research in Health UNSW Sydney Sydney Australia; ^3^ School of Graduate Studies and Institute of Policy Studies Lingnan University Tuen Mun Hong Kong; ^4^ L&E Research Consult Ltd, Upper West Region Wa Ghana; ^5^ The George Institute for Global Health Imperial College London London United Kingdom

**Keywords:** Benin, child, global health, morbidity, mortality, mass media exposure

## Abstract

**Introduction:**

Maternal exposure to mass media has been linked to various health outcomes, but its influence on childhood morbidity and mortality in Benin remains unexplored. This study examines the relationship between maternal mass media exposure and two critical child health indicators: morbidity and mortality.

**Methods:**

This secondary analysis of the 2017/2018 Benin Demographic and Health Survey (BDHS) included 13,851 women. Childhood morbidity (fever, cough, and diarrhea) and mortality were analyzed as outcomes. Bivariable and multivariable logistic regression and Cox regression models were applied, and results were expressed as odds ratios (OR), hazard ratios (HR), and their adjusted counterparts (aOR/aHR) with 95% confidence intervals (CI). Statistical significance was set at *p* < 0.05.

**Results:**

Fever was the most common morbidity (22.1%, 95%CI: 20.9–22.6), followed by cough (18.6%, 95%CI: 17.3–19.0) and diarrhea (13.1%, 95%CI: 12.6–14.1). At least one morbidity was reported for 35.7% (95%CI: 34.4–36.5) of children, whereas child mortality was observed in 5.6% (95%CI: 5.1–5.9). After adjustment, maternal exposure to radio and television was significantly associated with an increased likelihood of childhood cough and overall morbidity. Mothers with no exposure to radio (aOR = 0.76, 95%CI: 0.67–0.86) or television (aOR = 0.86, 95%CI: 0.75–0.99) had lower odds of their children experiencing cough compared to those exposed.

**Conclusion:**

Maternal exposure to mass media, particularly radio and television, was associated with increased childhood morbidity. This association could reflect heightened maternal health awareness, leading to increased reporting of symptoms or exposure to misleading health information through media content. These findings highlight the dual role of media as a tool for health education and a potential source of risk‐promoting influences. Policymakers and health practitioners should leverage mass media for targeted interventions while addressing its adverse impacts to improve child health outcomes.

## Graphical abstract

Maternal exposure to mass media, particularly radio and television, is linked to increased childhood morbidity in Benin. This may reflect both heightened health awareness and exposure to misleading information. Media can serve as a tool for health education, but its potential risks should be addressed to improve child health outcomes.

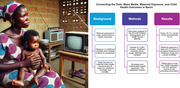

## 1 Introduction

Childhood morbidity and mortality remain pressing public health concerns in low‐ and middle‐income countries (LMICs), such as Benin, where infectious diseases like diarrhea, malaria, and pneumonia are the primary causes of these outcomes. In 2022 alone, about 4.9 million children under 5 died globally, translating to the death of approximately 13,400 children daily [1]. Although this statistic reflects global figures, it highlights the disproportionate burden borne by LMICs, where infectious diseases significantly contribute to these tragic outcomes. These deaths are largely attributable to morbid conditions, such as diarrhea, malaria, and pneumonia, among others [1]. Beyond mortality, several childhood ailments contribute to alarmingly high morbidity rates in Benin and other LMICs [2]. Hospitalization rates, long‐term health consequences, and the prevalence of childhood illnesses are notably driven by diseases like pneumonia, malaria, diarrhea, and vaccine‐preventable infections [3, 4].

Childhood mortality in Benin and other sub‐Saharan African countries is primarily driven by illnesses, such as diarrhea, malaria, and fever [5, 6]. Maternal socio‐demographic factors, including education, age, and wealth status, have also been linked to childhood morbidity and mortality [7, 8]. Although the UN Inter‐agency Group for Child Mortality Estimation (UN IGME) reported a decrease in child mortality in Benin from 98 per 1000 live births in 2015 to 84 per 1000 live births in 2021 [9], this rate remains far above the Sustainable Development Goal (SDG) target of 25 per 1000 live births, suggesting that Benin is unlikely to meet SDG target 3.2 [10].

The mass media, including print, radio, and television, is potentially useful for enhancing child health outcomes in resource‐constrained settings [11]. In Benin, radio remains the most widespread and accessible form of mass media, reaching a majority of the population, especially in rural areas where literacy levels are low. Radio programming often includes health‐related content, such as public health campaigns, immunization drives, and information about preventing common childhood diseases. Television, although less accessible than radio, has a growing audience in urban areas. Health‐related television programs frequently discuss maternal and child health issues, though advertisements promoting unhealthy products are also common. Newspapers, on the other hand, have a limited reach and primarily cater to urban, literate populations. Although newspapers occasionally cover health topics, their role in disseminating health information is less significant compared to radio and television.

Some evidence suggests that mass media can influence how people seek healthcare, how care is offered to others, and the social norms relating to children and holistic wellbeing [12]. The positive association between mass media exposure and improvements in childhood immunization rates and the use of oral rehydration therapy for the treatment of diarrhea has also been established [11, 13]. Existing evidence from sub‐Saharan Africa highlights the critical role of mass media in maternal healthcare. For instance, Aboagye et al. [14] realized a strong positive relationship between mass media exposure and maternal healthcare service utilization in a multi‐country study involving 28 sub‐Saharan African countries [14]. Similar findings have been reported from the sub‐region [15, 16].

Despite the plethora of literature on Benin children's morbidity and mortality [17, 18, 19], the association between mass media and childhood morbidity and mortality has not received sufficient scholarly attention. Although Yaya et al. [17] focused on the proximate and socio‐economic determinants of under‐5 mortality in Benin, Jerome et al. [18] assessed community health interventions for mitigating infant and child mortality in the southeastern part of the country. Relatedly, Gaffan et al. [19] assessed the relationship between access to water, sanitation, and hygiene (WASH) services and two childhood diseases: diarrhea and acute respiratory infection. There is, therefore, a knowledge gap regarding the association between mass media exposure and child morbidity and mortality in the Benin context.

Hence, we examined the association between mass media exposure and childhood morbidity and mortality in Benin. The study investigates the association between mothers’ exposure to television, radio, and newspapers and the incidence of childhood morbidity and mortality. The findings from our study have the potential to inform the design and implementation of media‐based interventions to enhance child health outcomes in Benin and other similar LMICs/settings.

## 2 Methods

### 2.1 Data Source and Target Population

This is a secondary data analysis of the 2017/2018 Benin Demographic and Health Survey (BDHS) [20]. This is the fifth version of BDHS, coordinated by l'Institut National de la Statistique et de l'Analyse Economique (INSAE) under the supervision of the Ministry of Planning and Development. Data for the 2017/2018 BDHS were collected between November 2017 and February 2018, with technical assistance from the Inner‐City Fund (ICF), International and financial assistance from other partners like the United Nations Children's Fund (UNICEF), United States Agency for International Development (USAID), the United Nations Population Fund (UNFPA), and the World Bank. Standardized procedures are followed in the aspects of questionnaire development, sampling procedure, data collection and cleaning, and other aspects. Data for this analysis were specifically from the women's file.

### 2.2 Sampling and Sample Size

A total of 15,928 women aged 15 and 49 years were surveyed during the 2017/2018 BDHS. The methodological approaches, including pretesting of questionnaire, training, and recruitment of enumerators inter alia, are detailed in the final report of the 2017/2018 BDHS [20]. In brief, the BDHS used a two‐stage sample design with the first stage involving cluster selection consisting of enumeration areas (EAs), and the second stage involved systematic sampling of households in all the selected EAs. Eligible women for the BDHS interviews were those aged 15–49 years and either permanent residents of the selected households or visitors who stayed in the household the night before the survey [20]. Given the scope of this study, we excluded women with no children under 5 years in their households and who had never given birth to a child in the last 5 years before the survey. As a result, 13,851 women were included in this analysis out of the 15,928 women interviewed during the survey.

#### 2.2.1 Outcome Variables

This study had two outcomes of interest, namely, childhood morbidity and childhood mortality. Childhood morbidity was measured as the reported history of three childhood illnesses (i.e., diarrhea, fever, and/or cough) in the 2 weeks preceding the survey. The three illnesses were selected because they are the commonly reported childhood diseases and had fewer missing values in the dataset. Besides, the selection of these three illnesses is consistent with existing literature that has focused on childhood morbidity in Benin and sub‐Saharan Africa [21, 22, 23, 24]. Childhood mortality, on the other hand, was measured as the reported history of childhood death (death under age 5) of the most recently born child before the survey in a household.

### 2.3 Independent Variables

The main independent variable in this study was maternal exposure to mass media. In the BDHS, this was measured by asking women their frequency of listening to radio, watching television, and reading newspapers or magazines, with response options 0: not at all, 1: less than once a week, 2: at least once a week, and 3: almost every day. These were re‐coded to 0: no media exposure (not at all) and 1: media exposure (for those with less than once a week, at least once a week, and almost every day). The same approach has been used in similar mass media studies [8, 24, 25].

### 2.4 Covariates

Several covariates were included on the basis of their well‐documented associations with childhood morbidity and mortality, as supported by existing literature [26, 27, 28]:

- **Age (15–24, 25–34, 35–44, 45 and above)**: Maternal age plays a critical role in child health outcomes, with younger and older mothers at higher risk of adverse outcomes due to biological, socio‐economic, and behavioral factors. For example, younger mothers may lack the experience or resources needed for optimal childcare, whereas older mothers may face higher pregnancy‐related health risks that can indirectly affect child health.
- **Marital status (married vs. unmarried)**: Marital status influences socio‐economic stability and caregiving capacity. Married women may have better access to resources and support systems, reducing risks of child morbidity and mortality.
- **Education level (no education, primary, secondary, tertiary, or above)**: Maternal education is a key determinant of health‐seeking behavior, including immunization uptake and utilization of healthcare services. Educated mothers are also more likely to practice preventive health measures and manage illnesses effectively.
- **Working status (working vs. not working)**: Employment status may affect a mother's ability to afford healthcare and maintain a hygienic environment, thereby influencing child health outcomes. However, it can also affect the time spent on caregiving.
- **Health insurance (yes vs. no)**: Access to health insurance enables families to afford necessary healthcare services, which is critical in preventing and managing childhood illnesses.
- **Parity (0, 1–2, 3–4, above 4)**: Parity influences maternal health and caregiving capacity. Higher parity may dilute resources and attention, increasing risks for child morbidity and mortality, whereas lower parity may reflect better health‐seeking behavior.
- **Healthcare decision‐making (yes vs. no)**: Mothers with greater autonomy in healthcare decisions are more likely to seek timely and appropriate medical care for their children, improving health outcomes.
- **Mode of childbirth (vaginal birth vs. caesarean birth)**: Caesarean births may be associated with complications that can affect child health, whereas vaginal births typically indicate fewer complications during delivery.
- **Household size (5 and below, 6 and above)**: Larger households may experience resource constraints, affecting child nutrition and healthcare access.
- **Household head (male vs. female)**: The gender of the household head influences resource allocation and decision‐making dynamics, with potential implications for child health.
- **Residence (urban vs. rural)**: Urban areas often have better healthcare infrastructure, access to clean water, and sanitation, reducing child morbidity and mortality risks compared to rural areas.
- **Problem with distance to a health facility (big problem vs. not a big problem)**: Distance from healthcare facilities is a critical barrier to accessing timely medical care, particularly in emergencies.
- **Type of toilet facility (improved vs. unimproved) and household water source (improved vs. unimproved)**: Access to improved sanitation and water sources reduces the risk of waterborne diseases, such as diarrhea, a major cause of childhood morbidity in LMICs.
- **Wealth index (poorest, poorer, middle, richer, richest)**: Socio‐economic status is a significant determinant of child health, as wealthier households are more likely to afford healthcare services, nutritious food, and improved living conditions.

These covariates were selected on the basis of their strong theoretical and empirical associations with childhood morbidity and mortality in sub‐Saharan Africa. This comprehensive inclusion ensures that the analysis accounts for potential confounding factors, enhancing the reliability of our findings.

### 2.5 Statistical Analysis

The analysis was performed with SPSS 26.0 (IBM Corp., Armonk, NY, USA) and involved bivariable and multivariable logistic and Cox regressions. The socio‐demographic characteristics were analyzed descriptively and presented as percentages and frequencies. The prevalence of the outcome variables (child morbidity and mortality) was compared according to maternal media exposure (radio, television, and newspaper), using chi‐square tests. Bivariable and multivariable logistic regression were used to examine the association of maternal media exposure and child morbidity. In contrast, Cox proportional hazards regression assessed the association of maternal media exposure and child mortality. In both regression models, relevant covariates were controlled for. The crude odds/hazard ratio (OR/HR), adjusted odds/hazard ratios (aOR/aHR), and their 95% confidence intervals (CI) are presented, with a *p* value <0.05 considered for statistical significance. The data were weighted to account for the survey's complex nature and multi‐stage sampling design.

## 3 Results

### 3.1 Characteristics of Participants

A total of 13,851 women were included in this analysis, and their background characteristics are listed in Table 1. The majority were below 35 years (68.1%), married (78.0%), working (78.6%), and had given birth at least thrice (55.9%), whereas almost all of them had no health insurance. About three in five participants (60%) had no formal education, two in five (41.6%) participated in healthcare decision‐making, three in five (61.3%) had a vaginal birth for their most recent baby, over 95% were from big households of six or more members, about four in five (79.1%) were from male‐headed households, and about three in five (60%) reside in rural areas. Distance to a health facility was not a problem to many (67.9%); over three in five (69.1%) used unimproved toilet facilities, and about a third (32.9%) lacked access to improved household water facilities (Table 1).

**TABLE 1:** Socio‐demographic characteristics of participants, from Benin Demographic Health Survey (*N* = 13,851).

Background characteristics	Weighted total sample % (*n*)
**Age**	
15–24	32.2 (4463)
25–34	35.9 (4986)
35–44	22.9 (3172)
45 and above	9.0 (1253)
**Marital status**	
Married	78.0 (10,817)
Unmarried	22.0 (3056)
**Education level**	
Tertiary or above	1.4 (198)
Secondary	19.3 (2674)
Primary	19.4 (2687)
No education	59.9 (8314)
**Religion**	
Catholic	23.0 (3188)
Islam	31.3 (4347)
Protestant	21.7 (3015)
Others	24.0 (3324)
**Working status**	
Working	78.6 (10,902)
Not working	21.4 (2971)
**Health insurance**	
Yes	1.0 (135)
No	99.0 (13,738)
**Parity**	
0	15.1 (2090)
1–2	29.0 (4027)
3–4	25.6 (3555)
Above 4	30.3 (4201)
**Healthcare decision‐making^1^**	
Yes	36.3 (5041)
No	41.6 (5776)
**Mode of delivery^2^**	
Normal virginal birth	61.3 (8501)
Caesarean birth	3.8 (529)
**Household size**	
Below 6	3.7 (513)
6 and above	96.3 (13,360)
**Household head**	
Male	79.1 (10,976)
Female	20.9 (2897)
**Residence**	
Urban	40.8 (5662)
Rural	59.2 (8211)
**Problem with distance to a health facility**	
Big problem	32.1 (4455)
Not a big problem	67.9 (9418)
**Type of toilet facility**	
Improved	30.9 (4288)
Unimproved	69.1 (9585)
**Household water source**	
Improved facilities	67.1 (9310)
Unimproved facilities	32.9 (4563)
**Wealth index**	
Poorest	18.3 (2545)
Poorer	19.5 (2702)
Middle	20.0 (2780)
Richer	21.2 (2948)
Richest	20.9 (2898)

*Note:* Missing values: ^1^ = 3056, ^2^ = 4843. Other religions included Vodoun, traditional sects, no religion, and others.

### 3.2 Prevalence of Maternal Mass Media Exposure, Childhood Morbidity, and Mortality

Of the 13,851 women in this study, 7.5% (95%CI: 7.2–8.1) were exposed to newspapers or magazines, 56.4% (95%CI: 55.4–57.0) to radio, and 36.3% (95%CI: 35.8–37.4) to television. The prevalences of outcome variables are detailed in Table 2. Regarding childhood morbidity, fever was the most common at 22.1% (95%CI: 20.9–22.6), followed by cough at 18.6% (95%CI: 17.3–19.0) and diarrhea at 13.1% (95%CI: 12.6–14.1). About 35.7% (95%CI: 34.4–36.5) reported at least one of the three morbidities among their children in the last 2 weeks before the survey. Child mortality was reported by 5.6% (95%CI: 5.1–5.9).

**TABLE 2:** Prevalence of childhood morbidity and mortality by maternal media exposure, from Benin Demographic Health Survey.

Variable	Overall weighted prevalence	Newspaper or magazine exposure^a^			Radio exposure^b^			Television exposure^c^		
		Yes	No	*p* value	Yes	No	*p* value	Yes	No	*p* value
**Diarrhea in last 2 weeks** ^d^				**0**.**043**			0.304			**0.025**
Yes	13.1 (1129)	10.2 (52)	13.3 (1076)		12.8 (611)	13.5 (518)		12.0 (358)	13.7 (771)	
No	86.9 (7490)	89.8 (460)	86.7 (7031)		87.2 (4176)	86.5 (3314)		88.0 (2633)	86.3 (4858)	
**Cough in last 2 weeks** ^d^				0.907			**<0.001**			**0.031**
Yes	18.6 (1599)	18.8 (96)	18.5 (1503)		20.6 (986)	16.0 (613)		19.8 (592)	17.9 (1007)	
No	81.4 (7020)	81.3 (416)	81.5 (6604)		79.4 (3801)	84.0 (3219)		80.2 (2398)	82.1 (4622)	
**Fever in last 2 weeks** ^d^				0.188			**0.013**			0.495
Yes	22.1 (1905)	19.7 (101)	22.3 (1805)		23.1 (1106)	20.9 (799)		21.7 (648)	22.3 (1257)	
No	77.9 (6714)	80.3 (412)	77.7 (6302)		76.9 (3681)	79.1 (3033)		78.3 (2342)	77.7 (4371)	
**At least one of the three morbidities** ^d^				0.168			**<0.001**			0.741
Yes	35.7 (3074)	32.8 (168)	35.6 (2881) 35.8 (2906)		37.6 (1799)	33.3 (1275)		35.4 (1059)	35.8 (2015)	
No	64.3 (5545)	67.2 (344)	64.2 (5201)		62.4 (2988)	66.7 (2557)		64.6 (1931)	64.2 (3614)	
**Death of a child** ^e^				0.361			0.225			0.194
Yes	5.6 (661)	4.8 (35)	5.7 (626)		5.8 (392)	5.3 (269)		5.2 (217)	5.8 (444)	
No	94.4 (11,122)	95.2 (698)	94.3 (10,424)		94.2 (6319)	94.7 (4803)		94.8 (3933)	94.2 (7189)	

Bold values significant at *p* = 0.05.

^a^ Newspaper or magazine exposure prevalence = 7.5% (1047).

^b^ Radio exposure prevalence = 56.4% (7827).

^c^ Television exposure prevalence = 36.3% (5030).

^d^ 5254 missing values.

^e^ 2090 missing values.

In comparison to women exposed to newspapers and television, those with no exposure reported significantly higher incidences of diarrhea (13.3% vs. 10.2% and 13.7% vs. 12.0%, respectively) among their children. However, women exposed to radio and television reported higher incidences of cough (20.6% vs. 16.0% and 19.8% vs. 17.9%, respectively) among their children compared to those with no exposure. Similarly, women exposed to radio, compared to those with no exposure, reported higher incidences of fever (23.1% vs. 20.9%) and at least one of the three morbidities (37.6% vs. 33.3%) among their children (Table 2).

### 3.3 Association of Maternal Mass Media Exposure and Childhood Morbidity and Mortality

Maternal exposure to newspaper or magazines was independently associated with childhood diarrhea (OR = 1.43, 95%CI: 1.06–1.92). Similarly, exposure to television had a positive independent association with diarrhea (OR = 1.20, 95%CI: 1.05–1.37) among under‐5 children. However, radio exposure showed negative independent associations with cough (OR = 0.74, 95%CI: 0.66–0.82), fever (OR = 0.89, 95%CI: 0.80–0.98), and having at least one of the three morbidities (OR = 0.83, 95%CI: 0.76–0.90) among children below 5 years (Table 3).

**TABLE 3:** Association between maternal mass media exposure and childhood morbidity and mortality.

Outcome variable	Crude odds ratio, OR (95%CI)	*p* value	Adjusted odds ratio, aOR (95%CI)^a^	*p* value
**Diarrhea**				
**Newspaper or magazine exposure**		**0.018**		0.963
Yes	1		1	
No	**1.43 (1.06–1.92)**		1.01 (0.72–1.42)	
**Radio exposure**		0.323		0.440
Yes	1		1	
No	1.07 (0.94–1.21)		0.95 (0.83–1.09)	
**Television exposure**		**0.008**		0.870
Yes	1		1	
No	**1.20 (1.05–1.37)**		0.99 (0.84–1.16)	
**Cough**				
**Newspaper or magazine exposure**		0.773		0.528
Yes	1		1	
No	0.97 (0.77–1.22)		0.92 (0.70–1.20)	
**Radio exposure**		**<0.001**		**<0.001**
Yes	1		1	
No	**0.74 (0.66–0.82)**		**0.76 (0.67–0.86)**	
**Television exposure**		0.062		**0.044**
Yes	1		1	
No	0.90 (0.80–1.01)		**0.86 (0.75–0.99)**	
**Fever**				
**Newspaper or magazine exposure**		0.146		0.797
Yes	1		1	
No	1.18 (0.94–1.48)		0.97 (0.74–1.26)	
**Radio exposure**		**0.024**		**0.045**
Yes	1		1	
No	**0.89 (0.80–0.98)**		**0.89 (0.80–0.98)**	
**Television exposure**		0.286		0.845
Yes	1		1	
No	1.06 (0.95–1.18)		0.99 (0.86–1.13)	
**At least one morbidity**				
**Newspaper or magazine exposure**		0.131		0.702
Yes	1		1	
No	1.16 (0.96–1.40)		0.96 (0.76–1.20)	
**Radio exposure**		**<0.001**		**<0.001**
Yes	1		1	
No	**0.83 (0.76–0.90)**		**0.80 (0.73–0.89)**	
**Television exposure**		0.532		0.190
Yes	1		1	
No	1.03 (0.94–1.13)		0.93 (0.82–1.04)	

Childhood mortality				
	Crude hazard ratio (95%CI)	*p* value	Adjusted hazard ratio, aHR (95%CI)^a^	*p* value
**Newspaper or magazine exposure**		0.387		0.256
Yes	1		1	
No	1.18 (0.85–1.65)		0.73 (0.42–1.26)	
**Radio exposure**		0.324		0.129
Yes	1		1	
No	0.92 (0.79–1.08)		0.84 (0.67–1.05)	
**Television exposure**		0.304		0.473
Yes	1		1	
No	1.09 (0.92–1.29)		0.91 (0.69–1.18)	

Bold values significant at *p* = 0.05.

^a^ The adjusted models controlled for age, marital status, education level, religion, working status, health insurance, parity, healthcare decision‐making, mode of delivery, household size, sex of household head, residence, problem with distance to a health facility, type of toilet facility, type of water source, and wealth index.

After adjusting for relevant covariates, maternal exposure to radio and television was significantly associated with the incidence of cough and at least one of the three morbidities. Mothers with no exposure to radio (aOR = 0.76, 95%CI: 0.67–0.86) and television (aOR = 0.86, 95%CI: 0.75–0.99) had less odds of reporting cough among their children. Similarly, mothers with no radio exposure (aOR = 0.80, 95%CI: 0.73–0.89) were less likely to report at least one of the three morbidities among their under‐five children compared to those with exposure (Table 3).

## 4 Discussion

Using a nationally representative sample, our study examined the association between maternal mass media exposure and childhood morbidity and mortality in Benin. Our study showed mixed results, as there were inconsistent prevalence rates of different childhood diseases depending on the type of media exposure. We found that women without exposure to newspapers and television reported higher incidences of childhood diarrhea, whereas women exposed to radio and television displayed higher incidences of cough among their children compared to those with no exposure. Similarly, women exposed to radio, compared to those with no exposure, reported higher incidences of fever and at least one of the three morbidities among their children.

As expected, we found that women who had newspaper and television exposure were less likely to report childhood diarrhea than their counterparts without such exposure. A likely explanation could be that women with such media exposure may acquire health information that positively influences their hygienic practices, thereby reducing the likelihood of their children contracting diarrhea pathogens. The plausibility of this relationship is further strengthened by the results of previous studies documenting how maternal media exposure improves health and developmental outcomes of their children [29]. Similar studies conducted in other LMICs, such as Nepal [30], Malawi [16], and the Democratic Republic of Congo [29], as well as in the broader sub‐Saharan African region [14], all illustrate a consensus on the positive association between media exposure and maternal and child health outcomes. It is important to stress that exposure to newspapers would significantly depend on mothers’ ability to access and comprehend information reported in these newspapers. Thus, maternal education, as supported by previous evidence [23], could be a critical factor influencing maternal media exposure.

Interestingly, we acknowledge that mothers with media exposure might have heightened awareness and reporting tendencies due to increased health‐related knowledge accessed through mass media. For instance, mothers exposed to health‐related content in radio and television programs might be more likely to recognize and report symptoms of childhood illnesses such as cough and fever. This potential bias may partly explain the higher prevalence of these illnesses among children of media‐exposed mothers, as observed in our findings.

In line with previous evidence focused on the adverse effects of media use on child outcomes [31], we also found that women exposed to radio and television were more likely to report childhood cough and fever among their children compared to women without exposure. The higher incidence of childhood cough and fever among children of mothers exposed to radio and/or television may also be attributed to the impact of commercial advertising on health‐related decisions and practices [32]. For example, research has shown that advertisements on television and radio often promote sugary drinks and unhealthy snacks, which can weaken children's immune systems, making them more susceptible to respiratory illnesses such as cough and fever [32]. Similarly, misleading over‐the‐counter medication advertisements may lead mothers to make inappropriate health decisions for their children, exacerbating these illnesses [33].

In an era of increased attention to social determinants of health, it is not uncommon for women with exposure to television and radio to access social advertising that impacts their maternal practices. This possibility is supported by an emerging body of research that has documented the harmful impact of commercial advertising on children's health‐related decisions and outcomes [32, 33].

Low or lack of maternal formal education may significantly impact mothers’ capacity to critically appraise potentially harmful commercial advertising. Our results showed that three in five of our participants had no formal education. With this high level of illiteracy, it may be practically impossible for mothers to assess potentially harmful information and/or commercial advertising they may be exposed to, thereby impacting their ability to make informed and scientifically accurate decisions. Previous studies have documented the negative effects of misleading information accessed via mass media on health and wellbeing decisions and practices [32, 34]. In corroboration of these studies, our findings suggest that such exposure—among a predominantly non‐formally educated sample—may be influencing health decisions and practices, likely explaining the higher incidences of childhood diseases such as cough and fever among women with mass media exposure compared to their counterparts without such exposure. This trend is similar to patters observed in other contexts, where limited education correlates with increased susceptibility to adverse health outcomes [35, 36].

To address these challenges, we recommend the promotion of media literacy programs to help mothers critically evaluate health information and identify potentially misleading content. Such programs can equip mothers with the skills needed to discern credible health‐related information and make informed decisions about their children's care. Additionally, we emphasize the need for regulating media content to prioritize accurate and beneficial health messages. Policymakers should enforce strict guidelines to ensure that media advertisements focus on promoting health‐enhancing products and services while discouraging the dissemination of content that may harm public health [35, 36].

Our study results highlight the importance of addressing both the benefits and unintended consequences of maternal mass media exposure. These findings underscore the need for integrated strategies that improve maternal education, regulate media content, and promote critical evaluation of health information to enhance child health outcomes.

### 4.1 Study Strengths and Limitations

The results of our study need to be understood within some caveats. Although the analysis is based on secondary data that is robust and representative, it is subjected to inherent limitations, including recall and social desirability biases. As a self‐reported survey, participants may have provided responses influenced by their perceptions of socially desirable behaviors, potentially leading to underreporting or overreporting of both mass media exposure and childhood morbidity and mortality [37].

Moreover, the mixed nature of our findings warrants careful interpretation. For instance, the higher prevalence of cough and fever among children of mothers exposed to mass media could reflect increased awareness and reporting tendencies due to greater access to health information, rather than a direct causal relationship. This potential confounding effect underscores the complexity of interpreting the observed associations and the possibility that increased reporting may inflate the apparent prevalence of illnesses among media‐exposed mothers.

Despite these limitations, our findings remain valuable for informing policies and interventions. They highlight the need to address both the potential benefits and unintended consequences of maternal mass media exposure, particularly in contexts where education levels are low and media content may not always be health‐promoting. Additionally, these results should serve as a catalyst for further primary research to explore the nuanced relationships between maternal socio‐economic and cultural characteristics, media exposure, and child health outcomes.

## 5 Conclusion

Our study revealed mixed results regarding the association between maternal mass media exposure and childhood morbidity and mortality. Although we found that women with mass media exposure were more likely to report higher incidences of childhood morbidities such as cough and fever, the interpretation of these findings should be approached with caution. It is possible that factors such as lower formal education or reporting biases, alongside potentially harmful content like commercial advertising, may contribute to these reported higher incidences among mothers exposed to mass media. Given the complexity of these associations, we suggest that addressing issues like girl‐child education and regulating media advertising on childcare standards could serve as important strategies to protect, improve, and promote child health outcomes in Benin.

## Author Contributions

S.Y. and J.K. contributed to the conceptualization of the study and executed the analysis and results write‐up with M.G. S.R.O. drafted Section 4. E.K.A. and S.Y. reviewed the literature and reviewed drafts for important intellectual content. S.Y. had final responsibility to submit. All authors read and amended drafts of the article and approved the final version for submission.

## Ethics Statement

This study is a secondary data analysis of BDHS; the authors had no encounter with the research participants, and so no additional ethical approval was required. However, we requested the dataset (https://www.dhsprogram.com/) and obtained approval to access and use the dataset for this study. Further information about Benin DHS data usage and ethical standards is available at the DHS website.

## Conflicts of Interest

The authors declare no conflicts of interest.

## Data Availability

The datasets analyzed during the current study are publicly accessible through the Demographic and Health Surveys (DHS) Program. Researchers and interested parties can obtain the data by visiting https://dhsprogram.com/data/available‐datasets.cfm. Access to these datasets requires registration and approval by the DHS Program to ensure compliance with their data use policy. The authors did not have any special access privileges to the data that others would not have.
